# *Yersinia pestis*: mechanisms of entry into and resistance to the host cell

**DOI:** 10.3389/fcimb.2013.00106

**Published:** 2013-12-24

**Authors:** Yuehua Ke, Zeliang Chen, Ruifu Yang

**Affiliations:** ^1^Institute of Disease Control and Prevention, Academy of Military Medical SciencesBeijing, China; ^2^Laboratory of Analytical Microbiology, State Key Laboratory of Pathogen and Biosecurity, Institute of Microbiology and Epidemiology, Academy of Military Medical SciencesBeijing, China

**Keywords:** invasion, anti-phagocytosis, intracellular survival, T3SS

## Abstract

During infection, *Yersinia*, a facultative intracellular bacterial species, exhibits the ability to first invade host cells and then counteract phagocytosis by the host cells. During these two distinct stages, invasion or antiphagocytic factors assist bacteria in manipulating host cells to accomplish each of these functions; however, the mechanism through which *Yersinia* regulates these functions during each step remains unclear. Here, we discuss those factors that seem to function reversely and give some hypothesis about how bacteria switch between the two distinct status.

## Introduction

Three *Yersinia* species are known to be pathogenic to humans: *Yersinia enterocolitis, Y. pseudotuberculosis*, and *Y. pestis*. *Y. enterocolitis* and *Y. pseudotuberculosis* are enteropathogenic bacteria causing enteritis, ileitis, and mesenteric lymphadenitis, whereas *Y. pestis* is the causative agent of bubonic plague, among the most deadly human infectious disease in history. All three species harbor a virulence plasmid, which encodes a type III secretion system (T3SS) for secreting Yop protein substrates, to establish a successful infection. Six Yops, including YopE, YopH, YopM, YopO/YpkA, YopP/J, and YopT, are delivered by T3SS into host cells and these then inhibit phagocytosis and block pro-inflammatory signals (Shao, 2008). In cases involving infection with enteropathogenic *Yersinia* species other than *Y. pestis*, two adhesins, invasin and YadA, have been shown to be important for mediating contact with host cells (Isberg et al., 1987; Paerregaard et al., 1991). However, although both adhesins are inactive in *Y. pestis* due to an IS1541 element insertion within *inv* and a frameshift mutation in *yadA* (Parkhill et al., 2001; Song et al., 2004; Chain et al., 2006), *Y. pestis* maintains the ability to attach to and enter into host cells (Davis et al., 1996; Perry and Fetherston, 1997; Cowan et al., 2000), indicating that these adhesins are not necessary for the virulence of *Y. pestis* and that other adhesins and invasins are required for mediating the association with host cells. *Y. pestis* is also known for its ability to survive in macrophages during its early invasion process. After arming itself in the macrophage, *Y. pestis* becomes resistant to phagocytosis and is then capable of surviving outside the cell, which is critical for its pathogenesis. In this review, we will summarize what is known regarding the mechanisms through which *Y. pestis* survives in a host, inside or outside the cell.

## Life cycle of *Y. pestis* during infection

*Y. pestis* is a facultative intracellular gram-negative bacterium. During the early stages of infection, *Y. pestis* can enter both macrophages and neutrophils through mechanisms of active or passive entry (Lukaszewski et al., 2005). However, *Y*. *pestis* is typically killed in neutrophils, whereas in macrophages, it can survive and acquire antiphagocytic capabilities, which enables its extracellular survival *in vivo* (Lukaszewski et al., 2005). Interestingly, *Y. pestis* can also enter into non-professional phagocytes, such as epithelial cells (Cowan et al., 2000; Leigh et al., 2005), which indicates that *Y. pestis* can not only be passively phagocytized by the professional phagocytes, but can also evolve a mechanism allowing it to invade host cells that generally do not possess phagocytic ability. The invasion of *Y. pestis* into host cells, including phagocytes and non-professional phagocytes, may be mediated by binding of adhesive factors present on their surface, including Ail, Pla, and Psa, to receptors on the membranes of host cells (Lahteenmaki et al., 2001; Miller et al., 2001; Benedek et al., 2004; Liu et al., 2006; Galvan et al., 2007; Felek et al., 2010).

**Figure 1:**
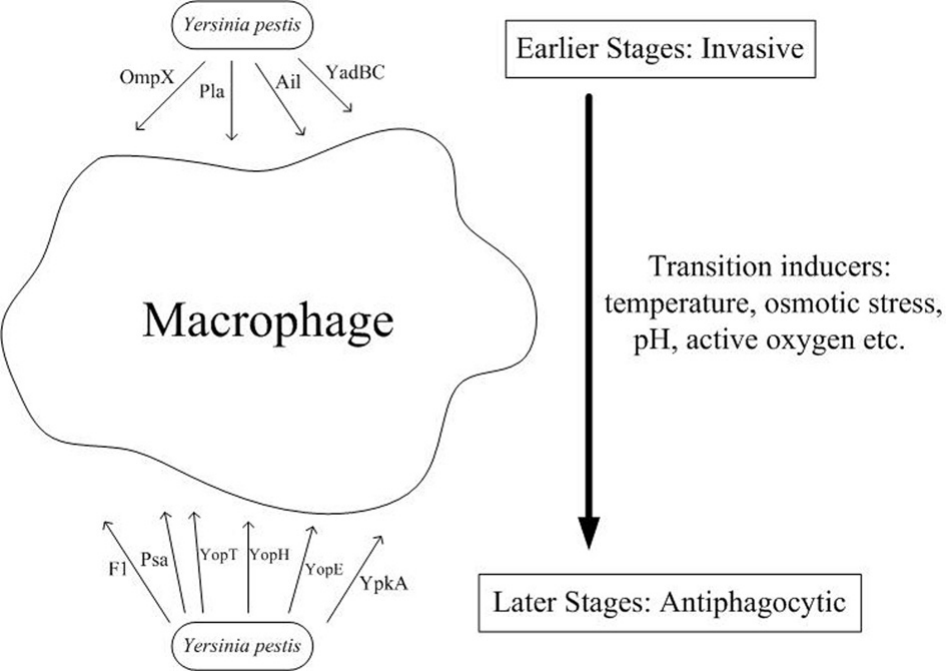
**Both invasive and antiphagocytic factors are involved in *Y. pestis* pathogenesis.** During the initial encounter with macrophages during infection, *Y. pestis* enters the macrophage through binding of its surface proteins, such as Pla, Ail, YadBC, and OmpX, to undetermined receptors present on the macrophage surface. However, following release from the macrophage through another undefined mechanism, *Y. pestis* expresses several virulence factors, including F1 antigen, Psa, and four Yops (YopT, YopH, YopE, and YpkA) which are employed to resist phagocytosis by the surrounding professional phagocytes. An additional mechanism subsequently arms *Y. pestis* against host phagocytes of the innate immune system, thereby allowing it to invade more tissues and organs and cause more severe impairment.

After arming itself inside the macrophages, *Y. pestis* escapes from the cell and develops resistance to phagocytosis by both macrophages and neutrophils. Therefore, during the late stage of infection, phagocytes cannot ingest *Y. pestis*, and the bacteria exists mainly in an extracellular environment, which has been confirmed through autopsies of human pneumonic plague victims, wherein the samples examined exhibited abundant extracellular bacteria but little evidence of phagocytosis. The mechanism of release from macrophages is largely unknown but may be associated with apoptosis or necrosis observed through *in vitro* cell models. Although the mechanism through which *Y. pestis* relocates from an intracellular compartment to an extracellular environment is unclear, the antiphagocytic ability of *Yersinia* has been attributed to Caf1, F1 antigen, and Yops (e.g., YopH, YpkA, YopE, and YopT) (Figure 1). Many *in vitro* studies have demonstrated that these virulence factors act synergistically to promote evasion or inhibit ingestion by host cells, including professional phagocytes and some non-professional phagocytes.

*Y. pestis* carries both invasive factors, which promote contact with and entry into host cells, and antiphagocytic factors that inhibit uptake by host cells. The mechanism through which this bacterium balances these contradictory factors and utilizes them in the different stages of infection to manipulate host cells remains an interesting topic. Here, we have discussed all of the known factors used by *Y. pestis* to enter into or escape from host cells.

## Phagocytic receptors of host cells and invasive ligands of bacteria

The process of bacterial entry into host cells can be divided into two types. The first type, bacterial invasion, involves active bacterial entry, during which bacteria are not welcomed and grudgingly accepted by the host cells. Many bacteria initially depend more on their own invasive factors to enter into the host cells in order to escape the harsh extracellular environment conditions, including low pH and shear stress, and host defensive mechanisms, such as recognition and killing by macrophages and cytotoxic T cells, thereby allowing them to survive in the relatively mild intracellular milieu. The second type, phagocytosis of bacteria, involves passive entry during which bacteria are resistant to internalization and are initially ingested by the host cells through a surface mechanism. Phagocytosis plays important roles in reducing the threat from bacterial pathogens; therefore, it is used by host cells to degrade and clear hazardous exogenous material, avoid disruption of normal functions by bacteria, and present signals for activation of the innate and adaptive immune systems. In some cases, these two types of entry are indistinguishable, as both modes of entry involve initial binding of bacterial ligands to phagocytic receptors (Greenberg, 1999). Although these two types of entry mechanisms are very popular in most bacterial pathogens and are easy to differentiate, some bacterial pathogens, such as *Mycobacteria* and *Coxiella*, utilize phagocytic signaling pathways to enter into the host cells, which may be hard to define their types of entry (Méresse et al., 1999; Friedrich et al., 2012).

Phagocytosis, classically defined as the cellular engulfment of particles larger than 0.5 μm in diameter (Jaumouille and Grinstein, 2011), is a highly conserved process that has evolved to counteract the threat of altered “self” molecular or “non-self” molecular. Phagocytosis encompasses an orchestrated cascade of events that involves particle recognition, signal transduction, cytoskeleton rearrangement, membrane remodeling, and phagosome maturation (Groves et al., 2008). In higher organisms, phagocytosis is mainly performed by professional phagocytes, such as macrophages, neutrophils, and dendritic cells (DCs), but several other cell types, including epithelial cells, endothelial cells, and fibroblasts, collectively identified as non-professional phagocytes, can also perform phagocytosis under certain conditions (Cannon and Swanson, 1992; Lowry et al., 1998; Sousa et al., 2005; Kim et al., 2010). Although non-professional phagocytes do not express FcRs and CRs, which are unique feature of professional phagocytes, the downstream molecular pathways of phagocytic receptors are generally expressed and once surface receptors are acquired, they can initiate the same phagocytic signaling pathway as professional phagocytes (Ezekowitz et al., 1991; Odin et al., 1991; van Zon et al., 2009).

The phagocytosis process begins with binding of a phagocytic receptor, directly or indirectly through opsonins, to their ligands on the surface of pathogens, which mediates the adherence of bacteria to the host cells (Pizarro-Cerda and Cossart, 2006; Huang et al., 2011). The best characterized phagocytic receptors are immunoglobulin receptors (FcRs) and complement receptors (CRs), which bind to bacteria, opsonized by antibody and complement respectively, and which activate downstream signaling pathways in a similar manner (May and Machesky, 2001; Groves et al., 2008; Nimmerjahn and Ravetch, 2008). A growing number of other cell surface receptors mediating phagocytic uptake of particles have been identified, such as scavenger receptors (e.g., SR-A, MARCO, CD36, and SR-B) and C-type lectins (e.g., mannose receptor, DC-SIGN, and dectin-1) (East and Isacke, 2002; Peiser et al., 2002; Cambi and Figdor, 2003; McGreal et al., 2005; Pluddemann et al., 2006; Vachon et al., 2006; Areschoug and Gordon, 2009). Additionally, members of the integrin family, excluding CR3, which belongs to the β2 subfamily, can also mediate entry of bacteria into host cells (Cambi and Figdor, 2003; Schraven and Reth, 2007). In particular, some receptors such as CD14 participate in the uptake of bacteria, but act only to tether the particles and cannot initiate phagocytic signaling alone, as this requires accessory receptors to deliver the phagocytic signal and co-start the phagocytic process (Wright et al., 1990).

A number of bacterial components, specifically ligands presented on their surface, are engaged during entry into host cells. Various components found on the plasma membrane of most gram-positive and gram-negative bacteria are likely involved in entry into host cells, including lipopolysaccharides, peptidoglycans, lipoteichoic acid, capsules, pili, and even CpG DNA (Pluddemann et al., 2006). In the past few years, a number of bacterial surface proteins have been shown to bind to the extracellular or surface receptors of the host cells or to be delivered into the cytosol of host cells, where they then assist in the invasive progress (Pluddemann et al., 2006; Areschoug and Gordon, 2008). The active entry of bacteria can be classified into two different mechanisms: “zippering” and “triggering” (Swanson and Baer, 1995; Cossart and Sansonetti, 2004). Zippering means that bacteria present ligands on their surface allowing them to bind to host cells and initiate the entry process (Cossart and Sansonetti, 2004). This zippering mechanism is exemplified by FcR- and CR3-mediated phagocytosis, which is characterized by the formation of inclusion shaped by the bacteria they ingest. Triggering is a mechanism through which bacteria inject effectors into host cells via T3SS to regulate phagocytosis (Swanson and Baer, 1995; Cossart and Sansonetti, 2004). The triggering mechanism enables host cells to internalize bacteria and fluids together (i.e., macropinocytosis) (Cossart and Sansonetti, 2004). For *Yersinia*, the zippering mechanism is the primary mode of entry since surface molecules of *Yersinia* mediate contact with, and adhesion of, host cells (Cossart and Sansonetti, 2004).

## Invasion factors

Bacterial pathogens can utilize many kinds of receptors present on the host cell surface, including the following, to facilitate invasion into host cells: FcγR; CR3; β1, β3, and β5 integrins; Toll-like receptors; mannose receptor; galactose receptor; and scavenger receptors (Taylor et al., 2005; Pluddemann et al., 2006). In addition to common strategies used by many other bacterial pathogens, *Yersinia* also employs several kinds of specific molecules on its outer membrane through which it can bind to and invade various host cells, including epithelial cells, endothelial cells, and fibroblasts, in addition to professional phagocytes. Indeed, *Y. pestis* has three identified adhesins: Ail (attachment-invasion locus), Pla, and Psa, in which Ail and Pla acts as invasive factors, but results of mediating adherence by Psa to host cells is not invasion, but prevention of invasion(as discussed below).

### Ail

Ail in *Y*. *pestis* is a 17.5 kDa outer membrane protein, encoded chromosomally, which is predicted to have eight transmembrane domains and four short extracellular loops extending from the surface of the bacterial outer membrane (Miller et al., 1990; Beer and Miller, 1992). Ail from all three species demonstrates cell adhesion and serum-resistance activities (Miller and Falkow, 1988; Miller et al., 2001; Kolodziejek et al., 2007; Bartra et al., 2008; Felek and Krukonis, 2009; Kolodziejek et al., 2010). In a mouse model of infection, Ail mutant *Y*. *pestis* exhibited a >3000-fold increase in the 50% lethal dose and a lower rate of colonization to the host tissue than the wild type (Felek and Krukonis, 2009).

In *in vitro* cell cultures, Ail-deficient strains have demonstrated reduced epithelial cell association and internalization, that is, at approximately 90 and 98%, respectively (Kolodziejek et al., 2007). *Escherichia coli* carrying Ail is highly invasive to CHO cells (Peiser et al., 2002) and moderately invasive to Hep-2 cells (Groves et al., 2008). Similar to YadA, Ail was also recently found to bind to fibronectin (Tsang et al., 2010), an extracellular matrix component. Additionally, treatment of cultivated cells with antifibronectin antibody decreased Ail-mediated adherence and inhibited KIM5-mediated cytotoxicity of host cells in an Ail-dependent manner (Tsang et al., 2010). Therefore, fibronectin, which has many integrin-binding sites, may act as a bridging molecular to engage host cells with this pathogen (Tsang et al., 2010). Moreover, because biochemical data and genetic analysis suggest that the C-terminal half of extracellular loop 2 of Ail mediates interaction with host cell surface components (Kirjavainen et al., 2008), it is possible that this domain interacts with fibronectin and is subsequently involved in attachment and entry into host cells.

Interestingly, despite sharing 100% homology with *Y. pestis*, Ail from *Y. pseudotuberculosis* fails to confer the same adhesion and invasion functions, which may be due to two amino acid substitutions in extracellular loop 3 (Yang et al., 1996; Miller et al., 2001; Tsang et al., 2010). However, Ail from *Y. enterocolitis* exhibits the same activity as that from *Y. pestis*, although they share only 70% sequence identity (Miller et al., 2001). These data provide strong evidence that some other factors contribute to Ail-mediated phagocytosis.

### Plasminogen activator

Plasminogen activator (Pla), encoded by the *Y. pestis*-specific plasmid pPCP1, is a membrane protein of the omptin family of bacterial outer membrane proteases (Kukkonen and Korhonen, 2004). Pla exhibits different phenotypes in inducing plague. In comparison with the wild type, *Y. pestis* lacking Pla has been reported to have greatly reduced virulence and was found to be one of the highly expressed genes when inoculated subcutaneously, but produced equivalent or nearly equivalent virulence when introduced by aerosols or directly into the blood stream (Sodeinde et al., 1992; Welkos et al., 1997). In models of pneumonic plague, dissemination of Pla-deficient *Y. pestis* to the circulation system was found to be unaffected, but restricted outgrowth was observed in lungs (Sebbane et al., 2006; Lathem et al., 2007). However, *Y. pestis* lacking Pla in mouse models of bubonic plague was observed to grow normally at the subcutaneous sites of inoculation but was not disseminated to the lymphatic system and deeper tissues (Sebbane et al., 2006; Lathem et al., 2007).

Pla is a 10-strand antiparallel β-barrel with four short periplasmic turns and has five surface-exposed loops where catalytic residues are located (Kukkonen and Korhonen, 2004). A striking feature of Pla is its ability to activate plasminogen to plasmin, which then dissolves fibrin clots and digests laminin that further impairs tissue barriers (Degen et al., 2007). In addition to proteolytic functions, Pla also plays a role in adhesion and invasion to epithelial cells, probably by binding to extracellular matrix components such as laminin and reconstituted basement membrane (Lobo, 2006). *Escherichia coli* expressing Pla is capable of invading HUVECs (Kukkonen et al., 2004), ECV304 (Lahteenmaki et al., 2001; Kukkonen et al., 2004), HeLa cells (Benedek et al., 2004), and macrophages (Zhang et al., 2008), and this process appears to be independent of residues S99 and D206, which are required for proteolytic activity of Plas (Lahteenmaki et al., 2001), thus providing evidence that the adhesion activity of Pla is independent of its protease activity. Pla-mediating internalization of bacteria by HeLa cells can be inhibited by phagocytic signaling inhibitors, such as wortmannin, staurosprin, genistein, C3 exoenzymes, and NDGA, and actin polymerization inhibitors, such as cytochalasin D, although bacterial association was not affected (Benedek et al., 2004). Additionally, the adhesion of Pla in *Y. pestis* is more efficient than that of its counterparts in other bacteria, such as PgtE of *Samonella*, OmpT of *Escherichia coli*, and Epo of *Erwinia* (Kukkonen et al., 2004).

Recently, Zhang et al. found that Pla is a ligand for a macrophage and DC surface receptor DEC-205, which is usually thought to be related to antigen presentation (Zhang et al., 2008). Using alveolar macrophage and CHO cells stably expressing DEC-205, the authors determined that Pla interacted with DEC-205 and that this interaction mediated adherence of *Y. pestis* to host cells and promoted early *Yersinia* dissemination from lungs to spleens during pneumonic plague (Zhang et al., 2008). These results suggested a new target of Pla activity, which may help elucidate the rapid progression of primary pneumonic plague.

### Other

YadBC, representing two surface proteins, is thought to be able to help bacteria to invade into WI-26 type1 pneumocytes and HeLa epithelioid cells (Forman et al., 2008). Although the YadBC double mutant of *Y. pestis* exhibited similar attachment to host cells, the invasion defect was small but significant (Forman et al., 2008).

## Antiphagocytic factor

### Psa

Psa, a homopolymer macromolecular complex known as an adhesion pilus, is assembled into a molecular mass of 15 kDa subunits, forming a capsule-like structure on the surface of *Y. pestis* (Lindler et al., 1990; Lindler and Tall, 1993). It is ideally expressed in an environment where the temperature remains at 37°C and pH ranges from 5.8 to 6.0 (Bichowsky-Slomnicki and Ben-Efraim, 1963). The expression of Psa is positively regulated by a global transcription regulator, RovA (Cathelyn et al., 2006) and is negatively regulated by Fur (Zhou et al., 2006). Mutated Psa is slightly attenuated by the intravenous mode of infection but exhibits a significant dissemination defect (Lathem et al., 2007). Recent studies have reported that the virulence of *Y. pestis* KIM5 lacking Psa was either unaffected or only slightly affected during subcutaneous challenge of Swiss Webster mice (Bearden et al., 1997) and was unaltered in native or immunized BALB/C mice (Anisimov et al., 2009).

Psa is known to bind to β1-linked galactosyl residues in glycosphingolipids (Payne et al., 1998) and apoB-containing LDL in human plasma (Makoveichuk et al., 2003), and these interactions could prevent binding of purified Psa to macrophages or fibroblasts (Payne et al., 1998; Makoveichuk et al., 2003). Indeed, Psa acts as a antiphagocytic factor independent of Yops (Huang and Lindler, 2004). Although Psa mediates the association of *Y. pestis* with human epithelial cells or mouse macrophages, it cannot resist uptake by host cells (Huang and Lindler, 2004; Liu et al., 2006; Grabenstein et al., 2006). In contrast to wild-type strains, Psa-mutated *Y. pestis* binding to human respiratory tract epithelial cells has been found to be significant, although the number of internalized bacteria was equivalent for both (Grabenstein et al., 2006). Additionally, Psa-modified *Escherichia coli* or polystyrene beads were observed to bind to host cells to a greater extent than either the control bacteria or beads, respectively, and their internalization by host cells was similarly poor (Huang and Lindler, 2004; Liu et al., 2006). These results indicated that Psa confers adhesive but not invasive activity to bacteria. Psa-mediating inhibition of phagocytosis may be caused by binding to lipoprotein, which could prevent recognition of bacterial pathogens by host cells.

### F1

Another fimbrial structure expressed by *Y. pestis* is fraction 1 (F1) antigen, which is composed of linear fibers of the Caf1 subunit (Zavialov et al., 2003). F1 is produced at high yields at 35–37°C to cover the bacterial surface. After the initial intracellular stage during infection, *Y. pestis* is released from macrophages and expresses large amounts of F1 (Du et al., 2002). Together with other anti-phagocytic factors, F1 efficiently limits phagocytosis of *Y. pestis* by host cells and contributes to the extracellular survival of *Y. pestis in vivo*, although F1-negative strains exhibit similar virulence as the wild type (Du et al., 2002).

A recent study provided direct evidence of the inhibiting effect of F1 on phagocytosis by epithelial cells (Liu et al., 2006). Using human respiratory tract epithelial cells as models, binding of F1-coated latex beads to A549 cells was reduced when compared with that to BSA-coated latex beads (Benedek et al., 2004). *Y. pestis* deficient in Caf1 was better internalized by host cells, and complementing this strain with Caf-containing plasmid significantly reduced internalization by host cells, confirming the F1-mediated inhibition of uptake of *Y. pestis* by respiratory tract epithelial cells (Benedek et al., 2004). Additionally, the authors of this study reported that the Caf-Psa double mutant was internalized more efficiently than the single mutant, thereby indicating that both F1 and Psa contribute to inhibition of phagocytosis (Benedek et al., 2004).

### YopH

YopH, a 468-amino-acid protein secreted by T3SS, exhibits the most potent activity of all PTPase(Protein Tyrosine Phosphatase) enzymes isolated to date (Zhang et al., 1992). The crystal structure of YopH indicates that its N- and C-terminal domains are linked by a proline-rich sequence. The N-terminal domain contains a type III secretion signal, a chaperon-binding region and a substrate-binding domain (Khandelwal et al., 2002), while the C-terminal domain includes a PTPase catalytic domain and an additional substrate-binding domain (Phan et al., 2003; Bahta and Burke, 2012). These two substrate recognition domains cooperate to reinforce binding of YopH to its substrate and enhance its activity and virulence.

The main function of YopH is anti-phagocytosis, which is executed by dephosphorylating its substrates, including p130^Cas^, FAk, and paxillin in epithelial cells and p130^Cas^, SKAP-HOM and Fyb in macrophages (Hamid et al., 1999; Deleuil et al., 2003; Aepfelbacher, 2004). These form the so-called focal adhesion complexes, which play pivotal roles in β1 integrin-mediating phagocytosis. By targeting and dephosphorylating these proteins, YopH may antagonize β1 integrin-mediated uptake of bacteria by host cells at an early stage. Antiphagocytosis activity is essential for the virulence of *Yersinia* since strains lacking YopH are efficiently ingested by host cell phagocytosis (Kerschen et al., 2004).

### YopE

YopE is a 219-amino-acid protein that exhibits eukaryotic GAP activity (Rosqvist et al., 1990; Black and Bliska, 2002; Pawel-Rammingen et al., 2002). YopE is comprised of three domains: an N terminal type III secretion signal followed by an intracellular membrane targeting domain and a C terminal Rho GAP domain (Black and Bliska, 2002; Pawel-Rammingen et al., 2002). In infected cells, translocated YopE has been found to cause cell rounding up and detachment through its ability to disrupt actin microfilaments (Black and Bliska, 2002; Pawel-Rammingen et al., 2002).

YopE inactivates small RhoA-like G proteins by inducing their GDPase activity, which results in conversion of the active GTP-bound state to an inactive GDP-bound state, and blocking of downstream signaling cascades (Aili et al., 2006). Given the importance of Rho GTPase in regulating actin polymerization, which is an important event in phagocytosis, YopE can antagonize phagocytosis of *Yersinia* by host cells (Viboud et al., 2006). Indeed, research has shown that YopE-deficient mutants can be efficiently internalized by macrophages and neutrophils (Songsungthong et al., 2010).

A recent study also demonstrated that YopE from *Y. enterocolitis* could bind to and inactivate RhoG, an upstream regulator of Rac1 and other Rho GTPases, both *in vivo* and *in vitro* (Roppenser et al., 2009). YopE colocalized with RhoG in the ER and Golgi, and this localization determined its substrate specificity and activity (Roppenser et al., 2009). As RhoG can be activated by the β1 integrin-mediating pathway of host cells when challenged by bacteria, inhibition of RhoG by YopE may contribute to the overall antiphagocytic activity in a new manner (Mohammadi and Isberg, 2009; Roppenser et al., 2009).

### YopT

YopT (322 amino acids) was identified as the second Yop, in addition to YopE, that can disrupt the actin cytoskeleton (Iriarte and Cornelis, 1998). YopT is similar to YopE in that its N-terminal region includes a type III secretion signal and a chaperon-binding domain (Sorg et al., 2003), although its C-terminus comprises a novel cysteine protease belonging to clan CE (Shao et al., 2003). YopT is expressed in *Y. pestis*, but in more than 50% cases, *Y. pseudotuberculosis* does not carry a functional YopT due to deletions in the region of the *yop*T gene (Schmidt, 2011).

YopT specifically recognizes and cleaves the phenyl groups of lipid-modified RhoA, Rac, and Cdc42, which results in detachment of Rho GTPase from the plasma membrane (Zumbihl et al., 1999; Shao et al., 2003). Mislocalization of Rho GTPase impairs its function and paralyzes various downstream signaling pathways (Iriarte and Cornelis, 1998). In cultured cells, ectopic expression of YopT leads to disruption of the actin cytoskeleton, cell rounding up, and inhibition of phagocytosis (Iriarte and Cornelis, 1998). Phagocytosis by macrophages and neutrophils of *Yersinia* mutants lacking YopT has been reported to be significantly greater than that by the wild type (Viboud et al., 2006).

However, the virulence of YopT-mutated *Yersinia* is not altered in mouse oral infection, and bacterial dissemination to the liver is not affected, suggesting that YopT is not required for virulence (Trülzsch et al., 2004; Mohammadi and Isberg, 2009). Additional studies have reported that YopT enhances virulence only in the absence of YopE (Viboud et al., 2006). Considering their similar activity, YopT may be a redundancy due to the presence of YopE, and this may partially explain why YopT is inactivated in certain strains.

### YpkA/YopO

YpkA (732 amino acids) is a multifunctional protein containing an N-terminal Ser/Thr kinase catalytic domain and C-terminal GDI domain (Navarro et al., 2007). The first 150 amino acids in the sequence comprise a type III secretion signal and membrane localization domain, while the last 20 amino acids comprise an actin-binding domain (Juris et al., 2000), which localizes to the inner face of the plasma membrane. YpkA is produced in bacteria as an inactive kinase and, when translated into host cells, is activated by binding to actin (Juris et al., 2000; Trasak et al., 2007). Upon activation, YpkA undergoes autophosphorylation, and then subsequently phosphorylates both native and artificial substrates, such as MBP and histone (Juris et al., 2000; Trasak et al., 2007).

Surprisingly, a recent study showed that the C-terminal domain of YpkA mimics the host GDI function to inhibit GDP-GTP exchange of small G proteins, like RhoA and Rac1, and to keep them in their inactive states (Prehna et al., 2006). This activity enables YpkA to repress Rho activity and to limit functions of its downstream signaling pathway, such as actin stress fiber formation (Barz et al., 2000; Dukuzumuremyi et al., 2000; Prehna et al., 2006). On the other hand, the kinase domain of YpkA can bind to and phosphorylate a G protein subunit G_α*q*_, thereby efficiently impairing guanine nucleotide binding and preventing activation of G_α_*q* (Navarro et al., 2007). Because both Rho proteins and G_α_*q* control actin stress fiber formation, simultaneous inactivation of these two targets enables YpkA to more efficiently usurp downstream signaling pathways of the host cells (Navarro et al., 2007).

Considering its obvious effect on the actin cytoskeleton, YpkA is believed to act as another important virulent factor, in addition to YopH, YopE, and YopT, in inhibiting phagocytosis of *Y. pestis* by host cells during infection (Wiley et al., 2006), as YpkA can sequester Rac and block Rac-dependent Fcγ receptor-mediated phagocytosis at the plasma membrane; however, this is not observed with RhoA and RhoA-dependent complement-mediated phagocytosis (Groves et al., 2010) as RhoA is trapped in the cytosol by endogenous RhoGDIα (Groves et al., 2010).

## Switch from invasive to antiphagocytic status

How does *Y. pestis* switch from the earlier invasive status to the later antiphagocytic status? After migrating to the inside of macrophages, *Y. pestis* bacteria would encounter harsher microenvironments, including higher temperature, low pH values, osmotic pressure, and reactive oxygen species (as shown in Figure 1). Consequently, *Y. pestis* may have evolved the ability to adapt to these challenges. Triggering by various kinds of environmental signals may have up- or down-regulated their survival- or virulence-associated genes, thereby changing surface components and presenting heterogeneous phenotypes. This transition may enhance their survival within host cells and their virulence once escaped.

First, and most importantly, the temperature shift from 28°C in vitro to 37°C in vivo could induce the expression of F1, Psa, and Yops, all of which work in coordination to block phagocytosis by host cells (Lawson et al., 2006; Fukuto et al., 2010). Second, the low pH value of the phagosome inside the macrophage allows for upregulation of the expression of PsA (Lawson et al., 2006; Fukuto et al., 2010). Although Psa mediates adhesion to host cells, this binding does not initiate the progress of uptake, and in contrast, inhibits the internalization of bacteria through an unknown mechanism(as discussed above). In addition, the Y.pestis-Containing Vacuole (YCV) may provide a niche for bacteria to make this transition. Around 8 h postinfection, the YCV acquired markers of late endosomes or lysosomes and had a spacious morphology, but it could prevent vacuole acidification and avoid bactericidal autophagy (Grabenstein et al., 2006; Pujol et al., 2009). Residence in the YCV and responses to environmental stress may promote bacteria to initiate the transcription regulation of invasive or anti-phagocytic factors, resulting into the final anti-phagocytic status.

Although the adhesive factors Ail and Pla are still present on the surface of bacteria, owing to their relatively short outer membrane regions, they are unable to escape the masking effects of the F1 capsule, which would block contact between Ail and Pla and their respective receptors, leading to further activation of the uptake signaling pathway. Conversely, the Psa is long enough to overcome the masking effects of the F1 capsule, but binding to its receptor does not prime the internalization progress and produces the opposite effect, which inhibits phagocytosis by host cells. Furthermore, adhesion of Psa to the surface of host cells facilitates the delivery of Yops into host cells, which further blocks the phagocytic ability of the host cells.

## Conclusions

Deficiency in invasin and YadA does not hamper the invasion of *Y. pestis* into host cells, which is necessary for infection during the earlier stage, which suggests that *Y. pestis* must utilize other invasive factors to aid its entry into host cells. Loss of virulence of these invasive-factor mutants indicates that *Y. pestis* must acquire the ability to tip the balance of entry into host cells and to subsequently have antiphagocytic activities. During the earlier stages of infection, *Y. pestis* requires invasive factors to enter into host cells, and during the later stages, it must produce antiphagocytic factors to escape uptake by host cells. Although the mechanism through which *Y. pestis* fine-tunes the temporal expression of these seemingly contradictory factors remains unknown, the simultaneous presence of these opposing functions in this bacterium represents a unique capability to survive both inside and outside host cells. Further analysis of the regulation of invasion and antiphagocytic associated virulence factors will improve our understanding of the pathogenesis of *Y. pestis* and will provide greater insights into the *in vivo* survival mechanism of facultative intracellular or extracellular bacteria.

### Conflict of interest statement

The authors declare that the research was conducted in the absence of any commercial or financial relationships that could be construed as a potential conflict of interest.
